# News and Events of the Week

**Published:** 1910-06-18

**Authors:** 


### News and Events of the Week.

The biennial dinner of Guy's Hospital will be held on Friday, July 1, at the Connaught Rooms, Dr. Hale White presiding.

Sir Alfred Keogh, K.C.B., LL.D., will distribute the prizes to students of St. Thomas's Hospital Medical School on Thursday, June 23, at 3 P.M., at the Hospital.

Dr. Amand Routh has been appointed the delegate of the Royal College of Surgeons to the Fifth International Congress of Obstetrics and Gynæcology to be held at St. Petersburg in September next.

A meeting of the Fellows of the Royal College of Surgeons of England will be held at the College in Lincoln's Inn Fields on Thursday, July 7, at 3 o'clock P.M., for the election of four Fellows into the Council of the College.

The Emperor William, accompanied by the Empress, opened last week, says the *Times* Berlin correspondent, the new quarters of the Emperor William Academy, a training college for the medical service of the German Army. The Academy, which is now situated in Invalidenstrasse, in the north of Berlin, contains, in addition to laboratories, a library, and other necessary educational adjuncts, 300 rooms for students.

An inquiry was recently held into the death of Miss Lyndal Rice, who was a student at the London (Royal Free Hospital) School of Medicine for Women, and had been found in the dining-room sitting in a chair dead. On a table beside her was a phial bottle, a glass, and a spoon. The bottle proved to be one in which a local chemist, Miss Spencer, had supplied Miss Rice with a tonic consisting of phosphate of iron. There were a few drops of a colourless liquid in the bottle, and Dr. Bell, not recognising the liquid, took the bottle to Miss Spencer and asked her to smell it. Instead of that she tasted it and immediately became greatly alarmed as to its effect upon herself, as she exclaimed, "This is not the tonic I gave her. This is prussic acid." Mr. Fisher, county analyst, considered that Miss Rice had taken a considerable quantity of prussic acid and that had caused death. Mr. Clarke, representing the friends, asked if it was not probable that cyanide had been taken and become converted into prussic acid by reaction in the stomach, but Mr. Fisher would not accept that view. Mr. Mudge, of the biological department of the School of Medicine, described the girl as one of a bright temperament and not likely to commit suicide. She was a keen student and did more than was really necessary with a desire to succeed in her studies. On one occasion she asked him the best way to kill a cat for laboratory purposes. The witness dissuaded her from using chloroform, and suggested the injection of cyanide of potassium into the cat's mouth by means of a glass syringe. He supported the suggestion that the presence of the glass and measuring spoon indicated that she had swallowed a dose of cyanide of potassium in mistake for the tonic, and that the cyanide became converted into prussic acid in the stomach. The jury returned a verdict to the effect that Miss Rice died from poisoning by prussic acid, but that there was no evidence to show where she obtained it or how it was administered. They also exonerated Miss Spencer from all blame, and were quite satisfied that she did not supply the poison.

The next vacation course under the auspices of the Berlin Dozeuten Verein will start on October 3 and will end on October 29 next. Full particulars of the course, which is open to all medical graduates, may be obtained on application to Herr Metzer, Ziegelstrasse 10-11 (Langenbeck Haus), Berlin, N.

Dr. John Finegan, otherwise O'Finegan, M.D., L.R.C.P.I., L.R.C.S.I., of Dublin, who died on April 24, left personal estate in the United Kingdom valued at £5,890. The testator left £160 for the celebration of Masses, and £100 for a stained-glass window in memory of his mother in St. Peter's, Drogheda.

The provincial sessional meeting of the Royal Sanitary Institute will be held in Trinity College, Dublin, at 8 P.M., on June 24, when discussions will take place on "The Water Supply of Irish Towns," to be opened by J. H. H. Swiney, M.Inst.C.E., and "The Bacteriological Standards of Potable Waters," to be opened by Prof. E. J. McWeeney, M.A., M.D. Tickets for admission of visitors may be had on application to Mr. W. Kaye-Parry, M.A., M.Inst.C.E., F.R.I.B.A., 48 Kildare Street, Dublin, who is kindly acting as the local hon. secretary of the meeting, and of E. White Wallis, secretary.

The National Association for the Prevention of Consumption and other Forms of Tuberculosis is to undertake a new educational campaign. A special appeal committee, of which Lord Derby is chairman and the Duke of Devonshire and Mr. Waldorf Astor are joint hon. treasurers, has been appointed to collect funds for the campaign, which is to be carried out by means of travelling tuberculosis exhibitions, caravans with lantern slides, popular lectures, an information bureau for the Press and public, and the distribution of leaflets. It should be added that 70,000 persons visited the Tuberculosis Exhibition in Whitechapel, and it was equally successful at Paddington, Marylebone, Walworth, Canning Town, Cambridge, and Oxford.

The President of the Local Government Board has authorised the following special researches: (1) A continuation of an investigation into protracted and recurrent infection in enteric fever, by Dr. Theodore Thomson, Medical Inspector of the Board, in conjunction with Dr. Ledingham, of the Lister Institute. (2) A continuation of an investigation into protracted and recurrent infection in diphtheria, by Dr. Theodore Thomson and Dr. C. J. Thomas. (3) A continuation of an investigation into flies as carriers of infection, by Dr. Monckton Copeman, Medical Inspector of the Board, in conjunction with Dr. Graham Smith and Mr. Merriman, of the University of Cambridge, Dr. Nicholl, of the Lister Institute, and Dr. Bernstein, of the Bacteriological Laboratory, Westminster Hospital. (4) A continuation of an investigation on the injurious gases evolved during artificial illumination, by Dr. J. Wade, D.Sc., of Guy's Hospital. (5) A preliminary inquiry into the relationship of certain special types of bacteria to the diarrhœa of infants, by Dr. C. J. Lewis, of Birmingham, Dr. Sheila M. Ross, of Manchester, Dr. Thomas Orr, of Shrewsbury, and Dr. R. A. O'Brien, of the Lister Institute.

At the Commemoration Day proceedings at Livingstone College, on Saturday, June 11, 1910, a reception was held to delegates to the forthcoming World Missionary Conference in Edinburgh, including many from the Continent of Europe and from America, sixteen British, eighteen Continental, and four American Societies being represented.

Prebendary Webb-Peploe having offered prayer, the Principal, Dr. C. F. Harford, after offering a cordial welcome to the delegates, explained that Livingstone College was in close touch with the great Missionary Societies of the world, and that many of these had sent representatives to Livingstone College. The name of Livingstone was honoured all the world over, and it was a matter of great regret to them that Dr. Livingstone's two daughters, Mrs. Livingstone Bruce and Mrs. Livingstone Wilson, both of whom had intended to be present, were prevented from attending by the sudden death of Mr. Livingstone Wilson at Sierra Leone less than a fortnight previously. He drew attention to the statement of the aims and objects of Livingstone College, which had been circulated in the Livingstone College Year Book, showing the vital necessity for medical training for those going abroad as missionaries, both for the preservation of their own health and for relieving the natives by whom they are surrounded, and who are cut off from any qualified medical assistance. Although Livingstone College students could not call themselves "medical missionaries," they knew how to apply their knowledge, whilst at the same time they recognised their limitations. He desired, above all, to emphasise the great importance of Livingstone College training as enabling students to join in the great campaign against malaria and other tropical diseases. To their great regret Professor Ronald Ross was prevented from being present, but he had sent a most important address indicating the ways in which missionaries could help to forward the propaganda which were being undertaken in different parts of the tropics. Herein he expressed the opinion that the missionary of to-day should, like the priest of old, be a healer both of the mind and of the body, and in addressing the meeting in commemoration of the greatest of explorers, travellers, and perhaps civilisers, he hoped that missionaries might again come to the front in connection with the remarkable development of science which had been witnessed during the last ten years in connection with malaria and other insect-borne diseases. He alluded to the great success that had attended anti-malarial measures, stating that the disease had been practically banished in Ismailia, Durban, Khartoum, Klang, and many other isolated spots, and that it had been greatly reduced in Italy, Panama, the Federated Malay States, and elsewhere. In conclusion, he strongly urged that every missionary who is likely to be stationed in a tropical country should go through a course of instruction at Livingstone College.

Dr. J. Howard Cook, a medical missionary of the Church Missionary Society in Uganda, who had lectured at the College for a short time before going abroad, described his experiences.

Mr. T. F. Victor Buxton, as chairman of the meeting, stated that he had known about Livingstone College since its foundation, and he had had an opportunity of seeing the work of some of the old students in Uganda, and he believed that the training was exactly what was needed, and he did not think that it could be regarded as trespassing on the prerogatives of qualified practitioners. He considered that the subscription list, which only amounted to £226 during the past year, was ludicrously small considering the work which was done, and he appealed for support to remove the deficiency of £265 on the last year's accounts and also to make possible permanent improvements which were in contemplation.

Dr. Olpp, who is a member of the staff of the Medical Missionary Institute at Tübingen, brought hearty congratulations from that Institute to Livingstone College. He said that he had seen the importance of the work done here, and that whilst half of the Institute at Tübingen is for training qualified medical missionaries, the other half is the same as Livingstone College—only made in Germany. The meeting closed with the Benediction, pronounced by the Rev. Canon McCormick.

---

The following appointments in the Royal Household have been gazetted :—

To be Physicians in Ordinary to His Majesty.—Sir Francis Henry Laking, Bart., G.C.V.O., K.C.B., M.D.; Sir James Reid, Bart., G.C.V.O., K.C.B., M.D.; Sir Richard Douglas Powell, Bart., K.C.V.O., M.D.

To be Physicians Extraordinary to His Majesty.—Sir Thomas Barlow, Bart., K.C.V.O., M.D.; Sir William Henry Allchin, M.D.; Bertrand Dawson, Esq., M.D.; Sir Alan Reeve Manby, M.V.O., M.D.

To be Physician to His Majesty's Household.—Sir Robert William Burnet, M.D.

To be Sergeant-Surgeons to His Majesty.—Sir Frederick Treeves, Bart., G.C.V.O., C.B.; Sir Richard Havelock Charles, K.C.V.O., F.R.C.S.I.

To be Honorary Surgeons in Ordinary to His Majesty.—Rickman John Godlee, F.R.C.S., M.S.; Anthony Alfred Bowlby, C.M.G., F.R.C.S.; Sir William Watson Cheyne, Bart., C.B., F.R.S.

To be Surgeon to His Majesty's Household.—Hugh Mallinson Rigby, Esq., F.R.C.S.

To be Surgeon-Apothecary to His Majesty and to His Majesty's Household.—Sir Francis Henry Laking, Bart., G.C.V.O., K.C.B., M.D.

To be Surgeon-Oculist to His Majesty.—Sir George Anderson Critchett, Bart., C.V.O., F.R.C.S.Ed.

To be Laryngologist to His Majesty's Household.—Milsom Rees, Esq., F.R.C.S.Ed.

To be Bacteriologist to His Majesty's Household.—Harold Robert Dacre Spitta, Esq., M.D.

To be Dental Surgeon to His Majesty's Household.—Charles Edwin Truman, Esq., M.R.C.S.

To be Anæsthetist to His Majesty.—Frederick William Hewitt, Esq., M.V.O., M.D.

To be Chemist and Druggist to His Majesty.— Mr. Peter Wyatt Squire.

To be Surgeons-Apothecary to His Majesty's Household at Windsor.—William Fairbank, Esq., M.R.C.S.; William A. Ellison, Esq., M.V.O., M.D.

To be Surgeon-Apothecary to His Majesty's Household at Sandringham.—Sir Alan Reeve Manby, M.V.O., M.D.

To be Surgeon-Apothecary to His Majesty's Household at Balmoral.—Alexander Hendry, Esq., M.D.

To be Honorary Physicians in Ordinary to His Majesty in Scotland.—Sir Thomas Richard Fraser, M.D.; David W. Finlay, Esq., M.D.

To be Honorary Surgeons to His Majesty in Scotland.—Alexander Ogston, Esq., M.D.; Sir William Macewen, F.R.S., M.D.

To be Honorary Surgeon-Oculist to His Majesty in Scotland.—George Andreas Berry, Esq., M.B.

To be Surgeon-Apothecary to His Majesty's Household at Holyrood Palace.—William Black Alexander, Esq.

To be Honorary Physicians in Ordinary to His Majesty in Ireland.—Sir Francis Cruise, M.D.; James Little, Esq., M.D.

To be Honorary Surgeons to His Majesty in Ireland.—Sir Charles Bent Ball, M.D.; Sir Thomas Myles, M.D.

To be Honorary Surgeon-Oculist to His Majesty in Ireland.—Charles Edward Fitzgerald, Esq., M.D.

